# ACE inhibition for severe bronchopulmonary dysplasia – an approach based on physiology

**DOI:** 10.14814/phy2.13821

**Published:** 2018-09-05

**Authors:** Arvind Sehgal, Mohan B. Krishnamurthy, Megan Clark, Samuel Menahem

**Affiliations:** ^1^ Monash Newborn Monash Children's Hospital Melbourne Victoria Australia; ^2^ Department of Pediatrics Monash University Melbourne Victoria Australia; ^3^ Pharmacy Monash Health Clayton Melbourne Victoria Australia; ^4^ Emeritus Head Paediatric and Foetal Cardiac Units Monash Medical Centre Monash Health Melbourne Victoria Australia

**Keywords:** ACE inhibitors, arterial stiffness, bronchopulmonary dysplasia, echocardiography, remodeling

## Abstract

Premature infants have a high incidence of bronchopulmonary dysplasia (BPD). Systemic hypertension, arterial thickness and stiffness, and increased systemic afterload may all contribute to BPD pathophysiology by altering left ventricular (LV) function and increasing pulmonary venous congestion by lowering end‐diastolic compliance. This case series studied the usefulness of angiotensin‐converting enzyme (ACE) inhibition by measuring clinical and echocardiographic improvements in six consecutive infants with “severe” BPD unresponsive to conventional therapy. The range of gestation and birthweight were 23–29 weeks and 505–814 g, respectively. All required mechanical ventilation (including high‐frequency oscillation) and all but one were administered postnatal corticosteroids. Other treatments including sildenafil and diuretics made no clinical improvements. Captopril was started for systemic hypertension after cardiac and vascular ultrasounds which were repeated 5 weeks later. A significant reduction in oxygen (55 ± 25 to 29 ± 3%, two‐tailed *P* = 0.03) and ventilator requirements, and improved cardiovascular parameters were noted. This included a trend toward reduction in aorta intima media thickness [840 ± 94 to 740 ± 83 *μ*m, *P* = 0.07] and an increased pulsatile diameter [36 ± 14 to 63 ± 25 *μ*m, *P* = 0.04]). Improvements were observed for both systolic (increased LV output, 188 ± 13 to 208 ± 13 mL/kg/min, *P* = 0.046 and mean velocity of circumferential fiber shortening, 1.6 ± 0.2 to 2.5 ± 0.3 [circ/sec], *P* = 0.0004) and diastolic (decreased isovolumic relaxation time, 69.6 ± 8.2 to 59.4 ± 5 msec, *P* = 0.044) function which was accompanied by increased pulmonary vein flow. Right ventricular output increased accompanied by a significant lowering of pulmonary vascular resistance. These findings suggest that improving respiratory and cardiac indices (especially diastolic function) warrants further exploration of ACE inhibition in BPD infants unresponsive to conventional therapy.

## Introduction

Bronchopulmonary dysplasia (BPD) is the most common and the most important respiratory consequence of preterm birth with incidence of approximately 60% in infants’ ≤25 weeks gestational age (GA) (Chow 2015). In infants born <32 weeks GA (cohort of this study), the Australia and New Zealand Neonatal Network has traditionally defined BPD as “lung disease with an ongoing requirement for supplemental oxygen therapy or ventilation support – high flow oxygen, continuous positive airway pressure, or mechanical ventilation – at 36 weeks postmenstrual age (PMA) (Chow 2015)”. A consensus definition for BPD severity suggests that “severe” BPD implies the infant's requirement of ≥30% oxygen and/or positive pressure respiratory support (positive pressure ventilation or nasal continuous positive airway pressure) (Jobe and Bancalari 2001). Pulmonary hypertension (PH) is a known complication of severe BPD and is associated with an increased morbidity, evidenced by longer length of stay and increased duration of respiratory support together with a higher mortality (Khemani et al. 2007; An et al. 2010; Bhat et al. 2012). Recent data using six objective echocardiography (ECHO) indices described the incidence to be approximately 40% among infants with severe BPD (Revanna et al. 2017).

**Figure 1:**
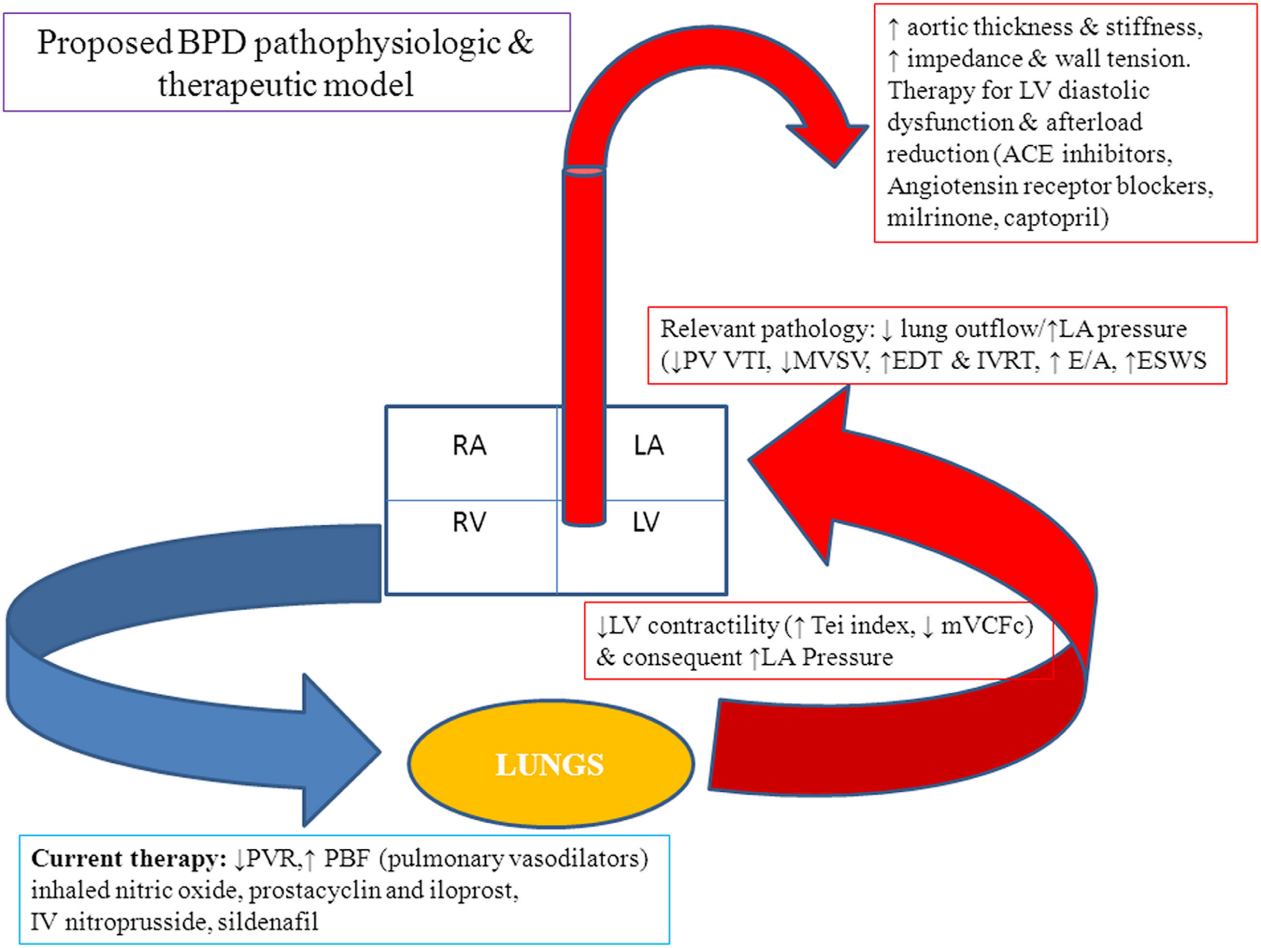
BPD pathophysiologic and therapeutic model (PVR – pulmonary vascular resistance, PBF – pulmonary blood flow, LV – left ventricle, LA – left atria, RV – right ventricle, RA – right atria, PV – pulmonary vein, VTI – velocity time integral, MVSV – mitral valve stroke volume, EDT – E wave deceleration time, IVRT‐isovolumic relaxation time, ESWS – end systolic wall stress, mVCFc – mean velocity of circumferential fiber shortening, ACE – angiotensin‐converting enzyme). With permission from SAGE journals. Sehgal et al. A new look at Bronchopulmonary dysplasia: Postcapillary pathophysiology and cardiac dysfunction. Pulm. Circ. 2016; 6:508–15.

Previous ECHO studies on BPD infants focused on the pulmonary aspects (right ventricular hypertrophy and the Doppler velocity of tricuspid regurgitation) (Khemani et al. 2007; An et al. 2010; Bhat et al. 2012). Treatment was directed toward PH, and included the use of pulmonary vasodilators such as inhaled nitric oxide (iNO) and sildenafil. The aim of such treatments was to reduce pulmonary vascular resistance, thereby increasing pulmonary blood in‐flow. Data on *systemic* (left sided) pathology were limited to the management of systemic hypertension. The incidence of systemic hypertension has been variously reported between 7 and 43% and was associated with longer duration of hospital stay, home oxygen, and a higher mortality (Abman et al. 1984; Anderson et al. 1993; Alagappan and Malloy 1998). An association between left ventricular (LV) hypertrophy and cerebrovascular accidents (Abman et al. 1984) has also been previously noted in infants with BPD. In a paradigm shift, investigators have recently focused on left‐sided cardiac *and* vascular changes which may be relevant to the pathogenesis and possible treatment of BPD (Sehgal et al. 2016a,b,c). A recent prospective study using vascular ultrasound noted increased arterial (aorta) wall thickness and stiffness in this population (Sehgal et al. 2016a,b,c). Its effects on the hypertrophy of the heart and reduced function consequent to increased afterload, and its likely contribution to BPD pathophysiology by way of pulmonary venous congestion has also been commented on in premature infants with BPD (Mourani et al. 2008 and Sehgal et al. 2016a,b,c). This previously published model, summarized in Figure 1, focused on the adjunctive role of back pressure changes from the altered systemic vasculature; LV hypertrophy, diastolic dysfunction, and raised LV end‐diastolic pressure, mediate this constellation of events (Sehgal et al. 2016a,b,c). This consideration, in a subset of BPD infants unresponsive to standard therapy, gains importance as conventional pulmonary vasodilators such as iNO and sildenafil may be ineffective or contraindicated.

Effects of ACE inhibitors other than blood pressure (BP) reduction (such as improved endothelial function, bradykinin‐mediated vasodilatation and endothelial NO release, and change in extracellular matrix) are physiologically very suited to the proposed BPD pathophysiology. We evaluated a series of consecutive infants with severe BPD unresponsive to standard therapy and systemic hypertension, using focused functional ECHO. The objective was to generate hypothesis regarding the use of ACE inhibitors that would need to be tested in appropriately designed randomized controlled trials.

## Methods

### Ethical approval

The study was approved by the Monash Health Research Ethics Board (approval number RES‐18‐0000‐249Q). The study conformed to the standards set by the latest version of Declaration of Helsinki, except for registration in a database. Standard practice in our Unit is to start captopril in consultation with renal and cardiac physicians after informed discussion with the parents when systolic BP is consistently above 99th centile for PMA (Dionne et al. 2012). Clinical data were retrieved from medical records. Infants were monitored using bedside ECHO (as part of standard management at the institution). All ultrasound recordings were performed using the Vivid 7 and E95 Advantage cardiovascular ultrasound system (GE Medical Systems, Milwaukee, WI, USA). Congenital heart disease and pulmonary vein stenosis were ruled out by ECHO. Offline analysis was performed using *EchoPAC™* (Horten, Norway). Standard ECHO indices were measured as described in detail previously (Sehgal et al. 2016a,b,c). Vascular and cardiac function indices were summarized as means ± SD. Data were analyzed using Stata software version 14 (StataCorp, College Station, Tex., USA) and SPSS version 18 (PASW Statistics for Windows; SPSS Inc., Chicago, Ill., USA). Variables were compared using Student's *t*‐test and statistical significance was regarded as two‐tailed *P* < 0.05.

### Unit protocol for the use of Captopril for systemic hypertension

Captopril is a competitive ACE inhibitor often used as a first‐line drug of choice in neonates with hypertension, heart failure, and congenital heart disease with left to right shunting or valvular regurgitation (Roche et al. 2016). Precaptopril investigations (complete blood count, serum electrolytes, urea and creatinine levels, plasma renin activity, aldosterone and cortisol levels, thyroid function tests, and head, renal and cardiac ultrasound) were performed and were within normal limits. BP was taken with the infant either asleep or in quiet wakeful state using an appropriate sized cuff, always in the same extremity (right arm). To avoid significant first‐dose hypotension, a low first dose is used before up titration to the target dose (Pereira et al. 1991; Roche et al. 2016; Rossi 2018; Taketomo et al. 2018). The initial dose was 0.01 mg/kg/dose oral every 8 h and was increased to 0.1 mg/kg/dose by the end of the first week. Subsequent increases were at 5–7 day intervals to reach the maximum dose of 0.5 mg/kg/dose, guided by systolic BP. The time to peak antihypertensive effect is 60–90 min after ingestion of captopril, however, the time to optimal antihypertensive effect following regular administration at a therapeutic dose is many weeks after initiation (Kubo and Cody 1985; Pereira et al. 1991; Taketomo et al. 2018). The first ECHO was done precaptopril. We made an a priori decision to repeat clinical and ECHO assessments after an interval of approximately 5 weeks based on the available information. This period would allow the infants to reach the target maximum dose and accumulate captopril metabolites, once the maximum dose was reached. While data in hypertension during infancy are limited, pharmacokinetic parameters for captopril in infants with congestive heart failure are within the range reported for adults with congestive heart failure (Pereira et al. 1991).

## Results

Table 1 summarizes the cohort characteristics. The GA and birthweight ranged from 23 to 29 weeks and 505–814 g, respectively. All but the sixth infant received antenatal corticosteroids, being delivered by an emergency caesarean due to an antepartum hemorrhage. All the infants in this series were managed with conventional and high‐frequency ventilation for respiratory distress syndrome. All but the second infant were administered surfactant. The Unit practice for postnatal corticosteroids for severe lung disease includes a low‐dose dexamethasone therapy (0.075 mg/kg/dose 12 h for 3 days then, 0.05 mg/kg/dose 12 h for 3 days then, 0.025 mg/kg/dose 12 h for 2 days then, 0.01 mg/kg/dose 12 h for 2 days then cease). The range of corrected GA at the start of corticosteroids was 27–34 weeks. The duration between first dose of postnatal corticosteroid administration and the administration of captopril ranged from 7 to 27 weeks. All the infants were on concurrent diuretics (hydrochlorthiazide and spironolactone). Additionally, the first four infants were also administered sildenafil (maximum 8 mg/kg/day) for pulmonary artery hypertension with no clinical improvement; addition of captopril was associated with its gradual wean and stoppage. A significant drop in oxygen and reduced respiratory support requirements was noted in all infants, when assessed approximately 5 weeks after the commencement of captopril. The mean oxygen requirement decreased from 55 ± 25% to 29 ± 3%, two‐tailed, *P* = 0.03 and the mean partial pressure of carbon dioxide trended down from 73 ± 10.3 mm Hg to 61 ± 9.2 mm Hg, two‐tailed *P* = 0.06, not reaching statistical significance. The systolic BP decreased from >99th centile (>100 mm Hg in all cases) to between 50 and 95th centile for PMA at repeat assessment.

**Table 1:** Cohort characteristics

Variable	Infant 1		Infant 2		Infant 3		Infant 4		Infant 5		Infant 6	
	Time 1	Time 2	Time 1	Time 2	Time 1	Time 2	Time 1	Time 2	Time 1	Time 2	Time 1	Time 2
GA at birth (weeks)	23		29		27		23		27		24	
Birthweight (g)	505		592		814		513		730		710	
Apgar score at 5 min	6		9		9		6		8		6	
Gender	Female		Female		Male		Female		Male		Male	
GA at start of sildenafil and maximum daily dose	44 weeks 8 mg/kg		43 weeks 6 mg/kg		36 weeks 6 mg/kg		26 weeks 6 mg/kg		–		–	
GA at postnatal steroids	27		–		34		32		31		30	
Duration between 1st postnatal steroid dose & captopril (completed weeks)	27		–		7		15		14		9	
Corrected GA at start of captopril and reassessment (completed weeks)	54	59	53	58	41	44	47	51	45	50	39	44
Ventilation mode	NIMV	HF	CPAP	HF	NIMV	NIMV	NIMV	CPAP	CPAP	LF	CPAP	HF
MAP (cm)/L of water	13	9	8	8	14	14	14	10	8	300 mL/min	9	7
Oxygen requirement (%)	40	28	45	30	60	34	100	27	28	25	55	30
pCO2	68	56	70	55	79	77	91	67	67	58	65	53
Discharge ventilation	HF 14, FiO_2_ 0.26 × 18 h & LF 0.25 L/min × 6 h		HF 14, FiO_2_ 0.4 × 18 h & LF 0.25 L/min × 6 h		HF 15, FiO_2_ 0.25 × 18 h & LF 0.25 L/min × 6 h		HF 10, FiO_2_ 0.21 × 18 h & LF 0.2 L/min × 6 h		LF 0.1L/min FiO_2_ 0.25		LF 0.15 L/min FiO_2_ 0.25	

GA – gestational age, MAP – mean airway pressure, L – liters, pCO_2_ – partial pressure of carbon dioxide, HF – high flow, CPAP – continuous positive airway pressure, NIMV – noninvasive mandatory ventilation, FiO_2_ – fractional inspired oxygen, LF – low flow.

Table 2 summarizes the vascular and ECHO measures before and approximately 5 weeks after captopril. The ductus arteriosus was closed in all cases. Two‐tailed analysis noted a trend toward reduction in aortic intima media thickness with a significant improvement in dynamic function (pulsatile diameter [beat to beat variation between systole and diastole]). Cardiac measurements noted improved contractility and output (systolic), and relaxation (diastolic function). Improved LV diastolic function maybe important as easing of LV end diastolic pressure was associated with improved trans‐mitral and pulmonary venous flow. While no other changes to medication profile were made during the period, right ventricular output increased, accompanied by an increased time to peak velocity/right ventricular ejection time (a noninvasive surrogate for reduction in pulmonary vascular resistance).

**Table 2:** Echocardiographic changes

Variable	Assessment 1	Assessment 2	*P*
Heart rate (bpm)	146 ± 12	152 ± 10	0.3
Vascular parameters			
Aorta intima media thickness (*μ*m)	840 ± 94	740 ± 83	0.07
Pulsatile diameter (*μ*m)	36 ± 14	63 ± 25	0.04
Left sided (systolic)			
Mean velocity of circumferential fiber shortening (circ/sec)	1.6 ± 0.2	2.5 ± 0.3	0.0004
Left ventricular output (mL/kg/min)	188 ± 13	208 ± 13	0.046
Left sided (diastolic)			
End systolic wall stress (g/cm^2^)	76.4 ± 5	58 ± 6.6	0.001
Trans‐mitral E/A ratio	1.1 ± 0.08	0.92 ± 0.06	0.003
Isovolumic relaxation time (msec)	69.6 ± 8.2	59.4 ± 5	0.044
Mitral valve stroke volume (mL/kg)	4.72 ± 0.2	5.46 ± 0.4	0.004
Pulmonary vein velocity time integral (cm)	5.3 ± 0.2	6.6 ± 0.6	0.002
Combined			
TDI Myocardial performance index	0.36 ± 0.03	0.28 ± 0.02	0.001
Right‐sided parameters			
Right ventricular output (mL/kg/min)	172 ± 9	193 ± 8	0.004
TPV/RVETc	0.27 ± 0.02	0.32 ± 0.02	0.008

TPV/RVETc – time to peak velocity/right ventricular ejection time (surrogate for pulmonary vascular resistance), TDI – tissue Doppler imaging.

## Discussion

The study of arterial biomechanics and left heart function in infants with BPD is relatively recent compared to well‐studied monitoring and management of PH (Mourani et al. 2008, 2009; Slaughter et al. 2011; Sehgal et al. 2016a,b,c). Systemic hypertension contributes to cardiovascular and respiratory morbidity. We noted that lowering of BP and improvement in *systemic* arterial measures (afterload reduction) using ACE inhibitors was associated with significant improvement in cardiac function and clinical features (respiratory support). Assessment of LV function (especially diastolic function) and the systemic vasculature may be warranted in infants with severe BPD with persistent radiological features of pulmonary edema despite diuretic therapy or where pulmonary edema worsens and respiratory support requirements increase, in response to the management of PH with pulmonary vasodilators. Such physiology‐driven approach toward therapy should be explored in infants with severe BPD and hypertension, especially in those unresponsive to standard therapy.

### Vascular pathophysiology in BPD infants: clinical relevance

**Figure 2:**
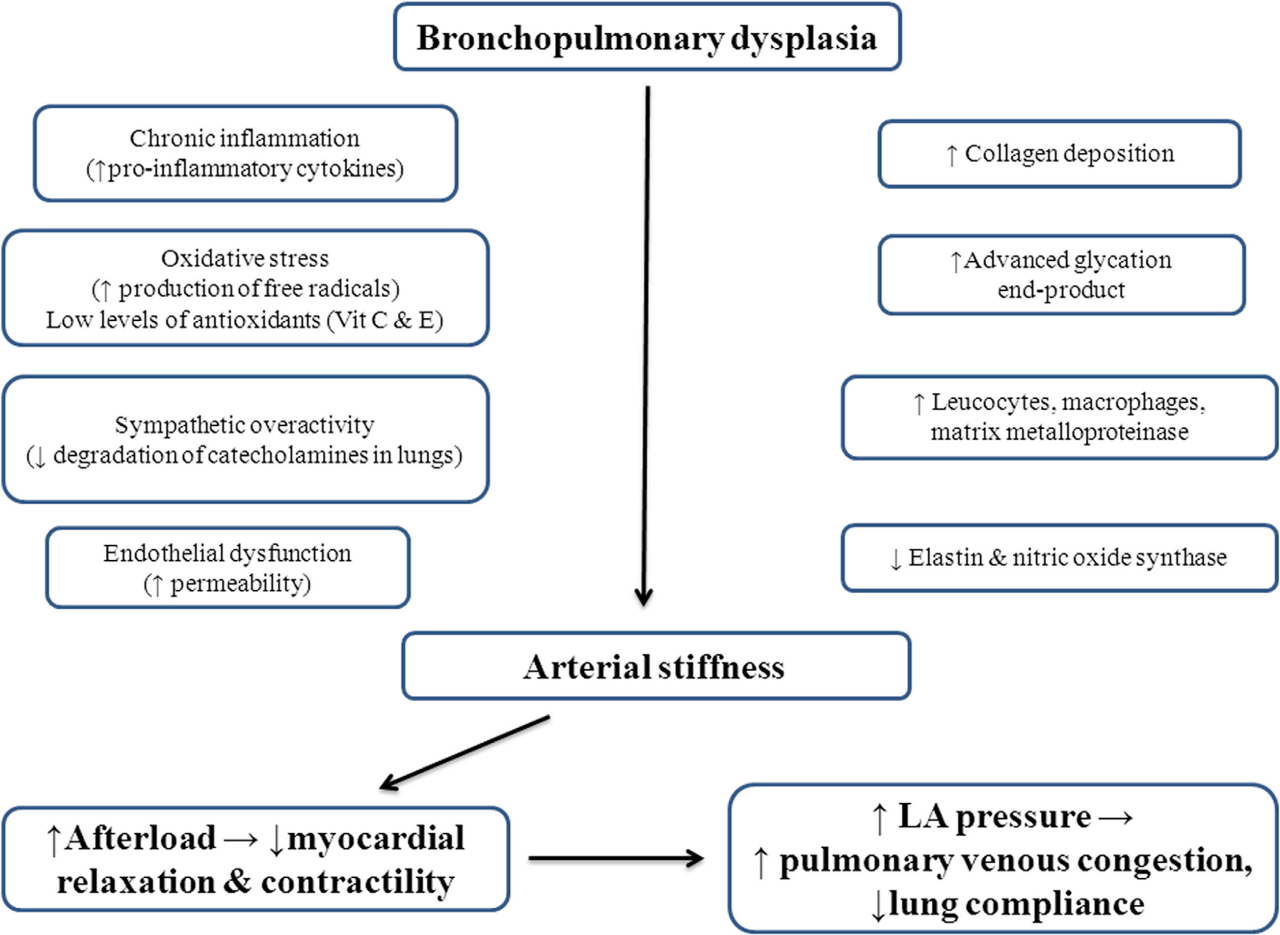
Postulated mechanisms of vascular/cardiac/pulmonary interaction in bronchopulmonary dysplasia (BPD). With permission from Springer Nature. Sehgal et al. Systemic arterial stiffness in infants with bronchopulmonary dysplasia: potential cause of systemic hypertension. J Perinatol. 2016; 36: 564–9.

Elevated proinflammatory cytokines, oxidative stress, and higher levels of catecholamines (increased sympathetic tone) have been implicated as possible mechanisms of systemic hypertension in this cohort (Mourani et al. 2009; Slaughter et al. 2011). We recently summarized key mechanisms mediating changes in arterial architecture and dynamics in infants with BPD (Sehgal et al. 2016a,b,c) (Fig. 2). Chronic inflammation is also associated with endothelial dysfunction (Vivodtzev et al. 2014). Impaired metabolism of catecholamines in the pulmonary circulation (impaired clearance and actual production) is a possible contributor (Abman 2000). The physiological synergism between catecholamines and arterial thickness suggests that ACE inhibition may be a particularly useful option in this cohort. The improvement in vascular and cardiac function parameters in our cohort after captopril mirrors previous data in adults and suggests benefits beyond BP reduction (Cacciapuoti et al. 1998; Higashi et al. 2000).

In a recent prospective study comparing preterm infants with severe BPD with GA matched preterm infants with no BPD and asymptomatic term infants, systemic arteries (abdominal aorta in this series) were found to be significantly thicker in BPD cases (Sehgal et al. 2016a,b,c). The dynamic function, characterized by impedance, stiffness and systemic vascular resistance, was significantly affected in the preterm BPD cohort. In adults, arterial stiffness is a strong predictor for cardiovascular events beyond that of classical risk factors such as pulse pressure (Boutouyrie et al. 2002). Evidently, the assessment of vascular properties is better positioned for longitudinal monitoring, compared to assessments of BP alone.

### Vascular effects of ACE inhibition beyond reduction in blood pressure

**Figure 3:**
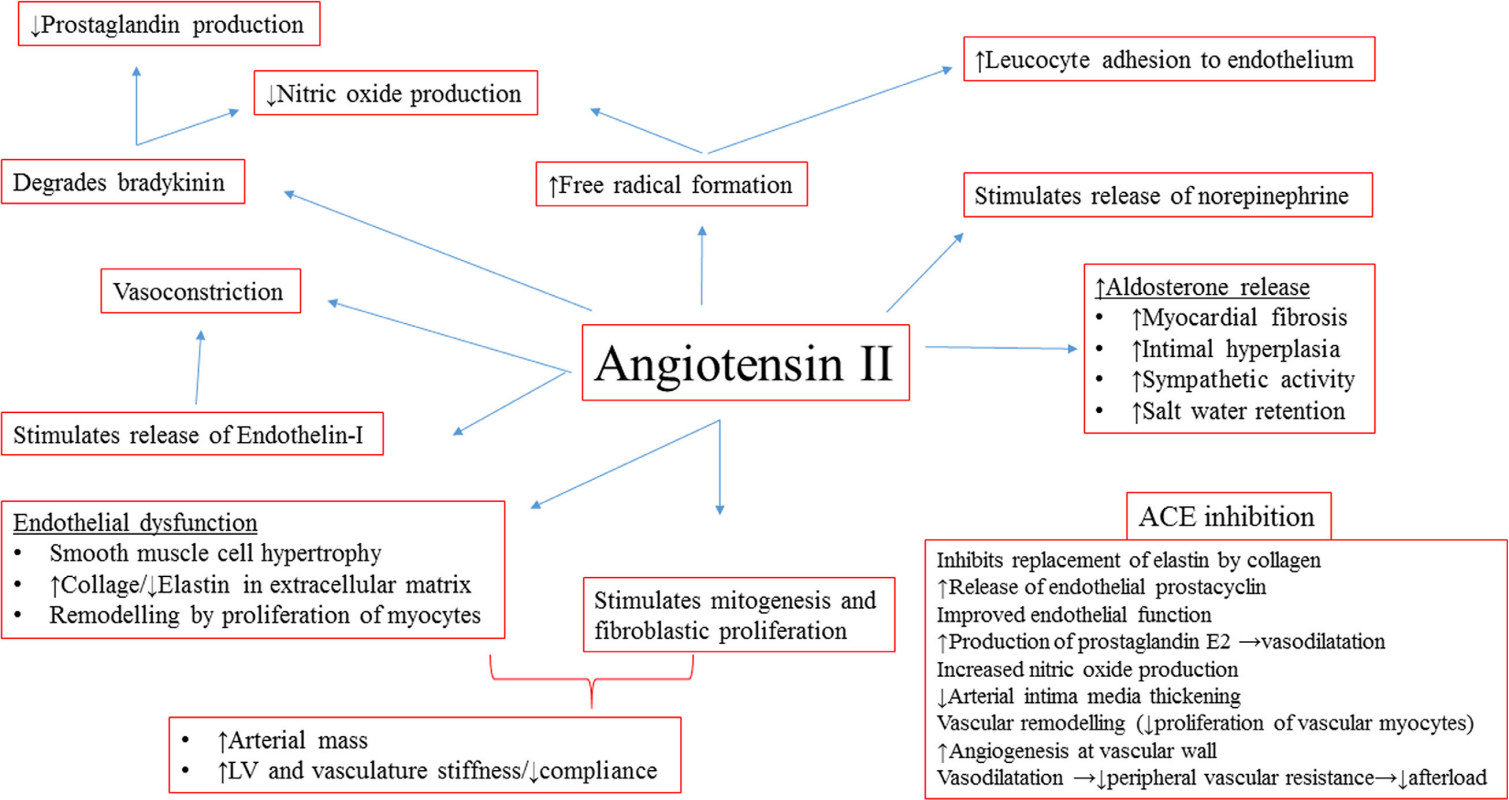
Mechanisms of action of angiotensin II on arterial vasculature: Role of ACE inhibition.

ACE inhibitors reset the balance between vasoconstriction/proliferation (Angiotensin II, reactive oxygen species, endothelin‐I) and vasodilatation/antiproliferation (bradykinin, NO, prostacyclin) of the vascular wall. Figure 3 summarize various roles of angiotensin II in disease physiology and the counter balancing mechanisms of ACE inhibition (Palmer et al. 1987; Rocchini et al. 1990; Linz et al. 1995; Schiffrin and Deng 1995; Gibbons 1997; Chrysant 1998; Miyajima et al. 1999; Weir and Dzau 1999; Anderson et al. 2000; Kawano et al. 2000; Bakris 2001; Lonn et al. 2001; O'Keefe et al. 2001; Schölkens and Landgraf 2002; Fenster et al. 2003; Fagard et al. 2009; Holecki et al. 2011). Two inter‐related mechanisms merit further discussion.

#### Endothelial function

Improvement in endothelial function regulates vascular smooth muscle tone and proliferation. This ability of ACE inhibitors to preserve and improve endothelial function is a significant advantage over other antihypertensive medications and of potential benefit in infants with BPD. In a multicenter study on 296 adult patients using strain‐gauge plethysmography, comparison was made between calcium antagonists, *β*‐blockers, diuretics, and ACE inhibitors. While all drug classes were equally effective in reducing BP, only the ACE inhibitors improved endothelial function and endothelium‐dependent vasodilation (Higashi et al. 2000).

#### Vascular wall remodeling

The blockade of the renin–angiotensin system has a significant impact on arterial structure and function *independent* of BP (Neutel 2004). Inhibition of arterial smooth muscle hypertrophy and the inhibition of the replacement of elastin fibers by collagen fibers in large arteries may mediate this (Chrysant 1998). Collagen has 100 times greater stiffness than elastin and leads to low arterial compliance/elevated afterload (Martyn and Greenwald 1997). In rats, preexposed to ramipril, antihypertensive treatment with ACE inhibitors (but not *β*‐blockers) caused regression of the media to lumen ratio in rats (Berkenboom 1998). Clinical human studies have now confirmed these finding with perindopril (Schiffrin and Deng 1995; Thybo et al. 1995; Zhuo et al. 1997). In contrast, treatment with a *β*‐blocker did not affect the abnormal vascular structure (Schiffrin and Deng 1995; Thybo et al. 1995). A prospective carotid ultrasound study on adults also noted reduction in intima media thickness with ramipril (ALLHAT Officers and Coordinators for the ALLHAT Collaborative Research Group 2002). Albeit in a smaller cohort and over a shorter time frame, our observational data mirrors these findings. The concurrent reduction in systemic afterload restores LV function (especially diastolic function) and reduces LV end diastolic pressure, encouraging forward mitral and pulmonary venous flow. The accompanying clinical effects of reduced pulmonary congestion were associated with significantly reduced oxygen and overall respiratory support requirements.

Given the angiogenic function of angiotensin‐II, ACE seems to play a role in vascular remodeling, pulmonary hypertension, and in the pathologic mechanism of neonatal lung diseases (Okoye et al. 1998; Lasaitiene et al. 2004; Yanamandra et al. 2004; Stenmark and Abman 2005; Kazzi and Quasney 2006). A recent histopathology review of all the autopsies with diagnosis of BPD over a 10‐year period noted that capillary ACE endothelial staining was largely absent compared to unaffected lungs (Castro et al. 2014). None of the BPD cases showed strong and diffuse staining seen with CD 31. This suggests that the improvements of requirements for respiratory support and vascular and ECHO parameters (especially pulmonary vascular resistance) in our cohort were primarily mediated via the *systemic* (rather than direct pulmonary) effects of ACE inhibition.

### Cardio‐protective effects of ACE inhibition

In our cohort, ACE inhibitors led to significant improvement in cardiac contractility/output and relaxation. A previous case report of two infants noted that assessments of LV diastolic dysfunction (and therapies tailored to improve that) may improve outcomes of infants with severe BPD, unresponsive to standard therapy with diuretics and pulmonary vasodilators (Mourani et al. 2008). While these infants were assessed by invasive cardiac catheterizations (a modality not widely available and possibly not without significant risks), vascular morphology and dynamics (the primary site of action of ACE inhibition) were not evaluated. Our study builds on this data using bedside ECHO assessments of LV function and vascular biomechanics. LV hypertrophy has been previously reported in infants with severe BPD (Mourani et al. 2008; Sehgal et al. 2016a,b,c). Efforts by the LV to generate ante grade flow by overcoming increased afterload and changes to extracellular matrix may possibly explain this. In effect, these LVs are muscular, but functionally suboptimal (reduced relaxation) which can lead to pulmonary congestion by way of increased mean LV end diastolic pressure. Evaluation of mitral valve Dopplers and pulmonary venous flow facilitates this assessment.

LV hypertrophy is a strong independent risk factor predicting sudden death, congestive heart failure and life threatening ventricular dysrhythmias (Messerli 1999; Mosterd et al. 1999); its regression improves these risks. The association of LV abnormalities with death in the setting of severe BPD mirrors these findings (Melnick et al. 1980; Abman et al. 1989; McConnell et al. 1990) and may be another factor supporting the use of ACE inhibitors. A randomized 3 year study on adults, comparing ramipril (ACE inhibitor) with felodipine (calcium channel blocker) noted that while both treatments produced a similar and significant reduction in BP, only ramipril prevented LVH; additionally improving systolic function in those patients with systolic dysfunction and heart failure (Cacciapuoti et al. 1998). Angiotensin II stimulates myocyte hypertrophy and increased formation of extracellular matrix (e.g., collagen) (Kawano et al. 2000); possibly explaining the efficacy of ACE inhibitors (Schmieder et al. 1996). The magnitude of this effect is substantial; one longitudinal study on adult patients noted this to be reduce LV mass by approximately 40% over the course of a 3 years, bringing it into the normal range (Franz et al. 1998).

## Conclusions

This consecutive case series of six infants with severe BPD included infants with systolic hypertension, managed with captopril. While small numbers as a limitation is accepted, this hypothesis generating study with detailed ECHO monitoring noted significant improvements in respiratory support and oxygen requirements and cardiovascular parameters. We acknowledge that these infants may have shown improvement over time in the absence of captopril treatment, but the combined improvement in clinical and ECHO parameters may be highly suggestive of genuine treatment effect.

While higher BP in infants with BPD is well recognized, the 99th centile threshold for making LV function assessments and initiating relevant therapy, may address only the tip of the iceberg. BPD infants with a higher BP (not exceeding the 99th centile threshold for antihypertensive therapy) are not routinely assessed using bedside ECHO. Assessments made much later in gestation (as in our cohort) probably mean prolonged duration of respiratory support and greater morbidity, longer duration of hospital stay and dedication of precious healthcare resources. As a future research direction for physiology‐driven care of this critically sick population, we propose that infants with severe BPD be assessed at close to 36 weeks corrected GA (GA at which the diagnosis of BPD is generally ascribed). Routine assessments of left heart function (especially diastolic function) and systemic arteries in addition to conventional right‐sided assessments will give greater insight into physiology‐driven care.

## Conflict of Interest

None of the authors has any conflicts of interests.
